# Immunotherapy Combined with Metronomic Dosing: An Effective Approach for the Treatment of NSCLC

**DOI:** 10.3390/cancers13081901

**Published:** 2021-04-15

**Authors:** Eleni Skavatsou, Maria Semitekolou, Ioannis Morianos, Theodoros Karampelas, Nikolaos Lougiakis, Georgina Xanthou, Constantin Tamvakopoulos

**Affiliations:** 1 Division of Pharmacology-Pharmacotechnology, Clinical, Experimental Surgery & Translational Research Center, Biomedical Research Foundation Academy of Athens (BRFAA), 11527 Athens, Greece; eleniskavatsou@gmail.com (E.S.); theodoros.karampelas@gmail.com (T.K.); 2 Cellular Immunology Laboratory, Center for Basic Research, Biomedical Research Foundation of the Academy of Athens, 11527 Athens, Greece; msemi@bioacademy.gr (M.S.); jmor@bioacademy.gr (I.M.); gxanthou@bioacademy.gr (G.X.); 3 Division of Pharmaceutical Chemistry, Department of Pharmacy, School of Health Sciences, National and Kapodistrian University of Athens, Panepistimiopolis Zografou, 15771 Athens, Greece; nlougiak@pharm.uoa.gr

**Keywords:** non-small cell lung cancer, metronomic chemotherapy, gemcitabine, oral agents, immunotherapy, efficacy, toxicity, pharmacokinetics, animal models, tumor microenvironment

## Abstract

**Simple Summary:**

Non-small cell lung cancer (NSCLC) claims almost 80% of the total lung cancer cases, with the late-stage disease having an estimated median survival time of up to five years. Patients with NSCLC benefit from traditional maximum tolerated dose (MTD) chemotherapy alone or combined with immunotherapy. However, efficacious such treatment options lead to side effects and poor patient quality of life. We show that metronomic (MTR) chemotherapy—based on the daily administration of chemotherapeutics in low, nontoxic doses—could potentially supplement MTD treatment options and indirectly prevent tumor growth leading to efficacy and less toxicity. Importantly when MTR chemotherapy is combined with an immunotherapy anti-PD1 agent, the anticipated efficacy is achieved with less toxicity, thus providing new options for the treatment of NSCLC.

**Abstract:**

Pioneering studies on tumor and immune cell interactions have highlighted immune checkpoint inhibitors (ICIs) as revolutionizing interventions for the management of NSCLC, typically combined with traditional MTD chemotherapies, which usually lead to toxicities and resistance to treatment. Alternatively, MTR chemotherapy is based on the daily low dose administration of chemotherapeutics, preventing tumor growth indirectly by targeting the tumor microenvironment. The effects of MTR administration of an oral prodrug of gemcitabine (OralGem), alone or with anti-PD1, were evaluated. Relevant in vitro and in vivo models were developed to investigate the efficacy of MTR alone or with immunotherapy and the potential toxicities associated with each dosing scheme. MTR OralGem restricted tumor angiogenesis by regulating thrombospondin-1 (TSP-1) and vascular endothelial growth factor A (VEGFA) expression. MTR OralGem enhanced antitumor immunity by increasing T effector responses and cytokine release, concomitant with dampening regulatory T cell populations. Promising pharmacokinetic properties afforded minimized blood and thymus toxicity and favorable bioavailability upon MTR administration compared to MTD. The combination of MTR OralGem with immunotherapy was shown to be highly efficacious and tolerable, illuminating it as a strong candidate therapeutic scheme for the treatment of NSCLC.

## 1. Introduction

Lung cancer constitutes the leading cause of cancer-related deaths worldwide, being responsible for an estimated 13% of cancer cases and 134,720 deaths for both sexes in 2020 in the US only [1]. NSCLC accounts for 80% of the total lung cancer subtypes. The majority of NSCLC patients undergo late diagnosis, leading to a short median survival time of up to 5 years as there are no efficacious treatments for metastatic stage IV NSCLC [2]. Many possibilities are being exploited to achieve the optimal therapeutic outcome, ranging from MTD chemotherapy, the prevailing chemotherapeutic model, to the more recently developed targeted therapies and immunotherapies. MTD chemotherapy needs extensive time breaks between each high dose to allow the patient to recover from the severe cytotoxic shock. Gemcitabine is an FDA-approved broad-spectrum deoxycytidine nucleoside analog used for NSCLC MTD treatment. A key limiting factor for gemcitabine’s efficacy stems from its metabolic properties. Following gemcitabine dosing, rapid deamination by cytidine deaminase leads to the production of the inactive metabolite, dFdU [3]. The MTD gemcitabine dosage and schedule for inoperable, locally advanced or metastatic NSCLC is a 28 day or a 21 day cycle (once per week), both schemes in co-administration with cisplatin [4]. Although MTD gemcitabine leads to cancer cell death, it exhibits serious toxicities. Importantly, the long intervals between treatments often allow tumor regrowth [5,6,7].

Developing effective anticancer therapeutic interventions requires a comprehensive understanding of the factors underlying tumor progression. Pioneering studies on tumor and immune cell interactions have uncovered immune checkpoint inhibitor (ICI) therapies as revolutionizing interventions for managing several cancers, including NSCLC. Binding of immune checkpoint receptors, such as programmed cell death ligand-1 (PD-L1) on cancer cells to programmed cell death protein-1 (PD-1) on T cells, inhibits T cell activation and induces a hypofunctional, “exhausted” T cell state that fails to contain tumor progression. ICI therapies block PD-1/PD-L1 interactions, reinvigorate T cell functions and allow the induction of destructive antitumor responses, leading to tumor eradication [7,8]. Although ICIs have been evaluated in NSCLC [8,9,10,11], with efficacy benefits over leading chemotherapeutics (e.g., docetaxel), adaptive resistance to PD-1/PD-L1 blockade has been documented [9]. Hence, current studies are focused on combination strategies involving chemotherapy and immunotherapy [10,11,12]. The clinical trial KEYNOTE-407 in which patients with metastatic, squamous NSCLC were cotreated with standard chemotherapy (carboplatin and either paclitaxel or nab-paclitaxel) and pembrolizumab, showed a prolonged median overall and progression-free survival [13]. Still, the toxicities of MTD chemotherapy remained present, illuminating the unmet need for developing targeted therapies that are more tolerable and efficacious.

Alternatively to existing chemotherapies that cannot distinguish between healthy and cancer cells, MTR chemotherapy consists of administering anticancer drugs in low-doses for an extended period, without long breaks, and targets peripheral mechanisms that affect tumor’s formation and progression. Clinical studies have uncovered the antitumor potential of MTR gemcitabine in advanced gallbladder and biliary tract carcinoma. Importantly, upon MTR dosing, gemcitabine’s concentration in plasma was low and well-tolerated [14,15,16,17]. However, MTR pharmacokinetic data have been reported only on a small group of drugs in animal models [18] and patients [19], limiting current knowledge on the therapeutic capacity of MTR chemotherapy. Considering that MTR chemotherapy necessitates daily administration of the anticancer agents, oral drugs or prodrugs are better suited to ensure patient compliance and well-being. OralGem (also known as LY2334737) is a prodrug of gemcitabine with satisfactory preliminary results when administered orally, with improved bioavailability and prolonged systemic exposure to gemcitabine [14,15,20]. In the long-term, MTR chemotherapy can offer significant benefits also in the field of health economics, as patients would be able to self-administer the drug without the need for hospitalization, decreasing healthcare costs.

In the present studies, we evaluated the effects of MTR therapy on NSCLC animal models through a systematic study of cancer and immune mechanisms, using OralGem as a well-suited prototype. Relevant in vitro and in vivo animal models, as well as state-of-the-art bioanalytical methodologies, were developed to explore various parameters of efficacy, bioavailability and toxicity and immunophenotyping of the tumor microenvironment (TME). Moreover, the co-administration of an anti-PD-1 neutralizing antibody was explored to evaluate whether this combination can improve the efficacy of MTR OralGem chemotherapy.

## 2. Results

### 2.1. MTD Gemcitabine Dosing Generates High Circulating Drug Concentrations Compared to MTR OralGem Administration

**Figure 1:**
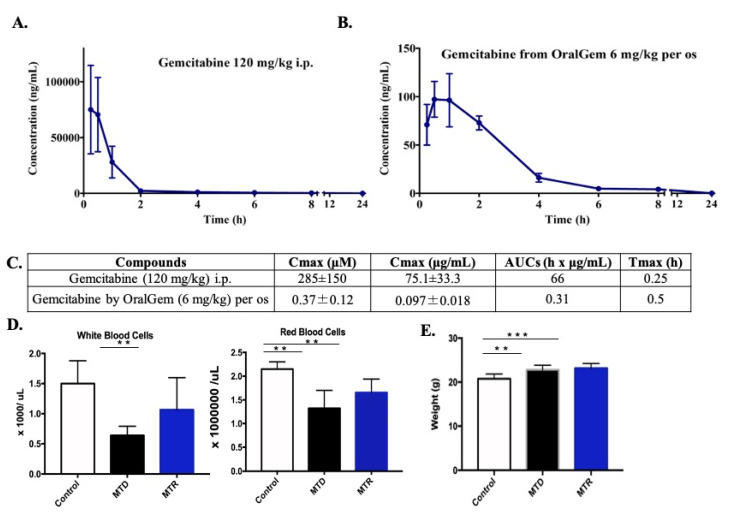
Pharmacokinetic and toxicity analysis of metronomic (MTR) OralGem treatment. (**A**) Gemcitabine was administered i.p. at the high dose of 120 mg/kg in C57BL/6 mice, reaching the concentration of 285 μM in circulating blood (*n* = 6, set of experiments = 1). (**B**) OralGem was administered orally once at a dose of 6 mg/kg in C57BL/6 mice, leading to the generation of gemcitabine in the bloodstream at a maximum concentration of 0.4 μM (*n* = 6, set of experiments = 1). (**C**) Table showing the C_max_, Area Under the Curve (AUCs) and T_max_ of each compound. (**D**) C57BL/6 wild-type mice were used and divided into 3 main groups. In the maximum tolerated dose (MTD) group, mice (*n* = 5) were administered intraperitoneally a dose of 120 mg/kg 8 times in a period of 21 days, whereas, in the MTR group, animals (*n* = 6) were administered orally the low dose of 6 mg/kg of OralGem every day for 21 days. Finally, the control group (*n* = 5) received orally 21 times carboxymethyl cellulose (CMC) 0.5% (set of experiments = 1). When the protocol was completed, mice were sacrificed, and whole blood was collected and analyzed to detect any alteration of white (WBCs) and red blood cells (RBCs). Numbers of WBCs and RBCs in both types of treatment (ΜTR *n* = 6, MTD *n* = 5) compared to the control group (*n* = 5). (**E**) Average weight (g) of animals. Data represent mean ± SD. Statistical analysis was performed by Student’s *t*-test; ** *p* < 0.01 and *** *p* < 0.001.

The pharmacokinetic profile of both OralGem and gemcitabine was evaluated in C57BL/6 mice. Following dosing of OralGem, the maximum blood concentration of gemcitabine was achieved at 0.5 h (C_max_ = 0.37 μM ± 0.12) with an AUC_0.25–24 h_ of 0.31 h × μg/mL. In contrast, when gemcitabine was administered i.p. at 120 mg/kg, the concentration of gemcitabine in circulating blood reached 285 μM ± 150 with an AUC_0.25–24 h_ of 66 h × μg/mL (Figure 1A,B). The MTD gemcitabine dosing generated approximately 700 times more circulating drugs in the blood compared to that generated after a low dose of OralGem (Figure 1C). These extremely high initial circulating levels following MTD administration can lead to sufficient bioavailability and efficacy but are also associated with severe off-target toxicities. A pharmacokinetic profile of prolonged systemic exposure, such as that of OralGem, would be more appropriate to ensure efficacy with lower circulating concentrations to avoid toxicities. Altogether, daily per os administration of OralGem leads to sustained plasma concentrations, which in turn may lead to efficacy and improved tolerability over the MTD scheme.

### 2.2. Decreased White Blood Cell Toxicity upon OralGem MTR Compared to MTD Administration

As gemcitabine causes severe blood toxicities, we next evaluated the potential safety advantages of MTR OralGem over MTD chemotherapy. Indeed, upon MTD chemotherapy, there was a significant decrease in the number of WBCs, compared to the control group, whereas MTR chemotherapy did not cause notable changes (MTD: 0.6 ± 0.2 × 1000/uL, MTR: 1.1 ± 0.5 × 1000/uL, control: 1.5 ± 0.4 × 1000/uL) (Figure 1D). Both types of chemotherapy induced a decrease in the RBCs compared to the control group, with the MTD treatment having the more pronounced reduction (MTD: 1.3 ± 0.4 × 10^6^/uL, MTR: 1.7 ± 0.3 × 10^6^/uL, control: 2.1 ± 0.2 × 10^6^/uL) (Figure 1D). During this period, animals were constantly weighted as another surrogate for chemotherapy toxicity, and no weight changes were observed between the MTD and MTR groups, with a slight increase observed in treated animals compared to the control group (Figure 1E). Overall, compared to conventional MTD gemcitabine administration, MTR OralGem treatment resulted in lower doses of circulating chemotherapeutic drug and exhibited decreased toxicity on WBCs, critical for the generation of antitumor immunity.

### 2.3. Enhanced Antitumor Efficacy, Associated with Reduced Angiogenesis upon MTR OralGem Administration

**Figure 2:**
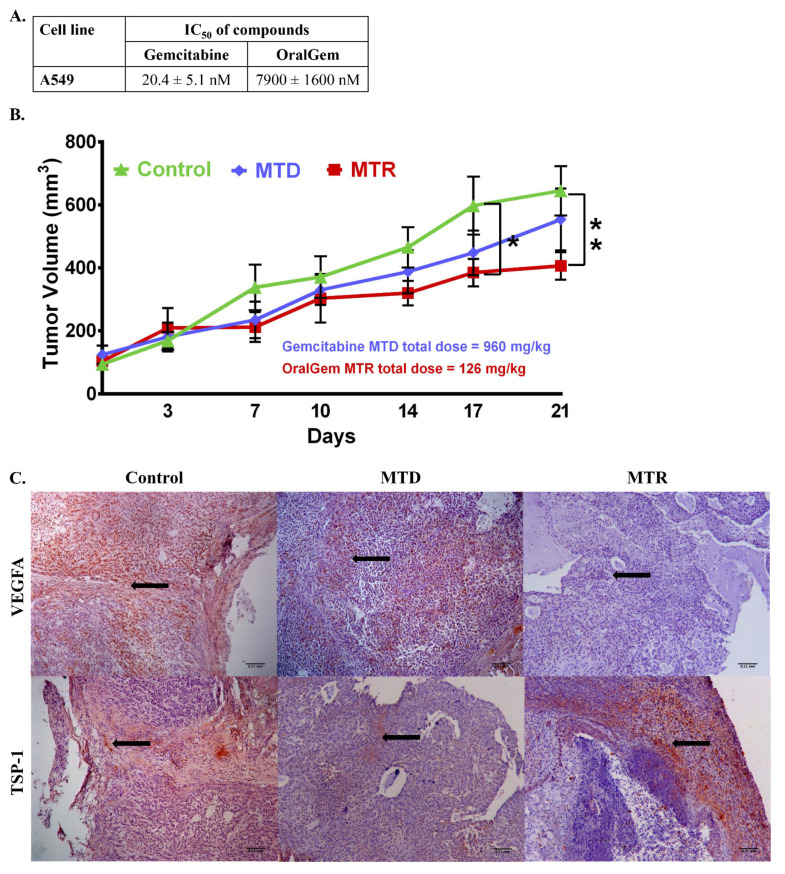
Investigation of efficacious dosing rounds of gemcitabine MTD and OralGem MTR in a xenografted mouse model. (**A**) IC_50_ values of gemcitabine and OralGem on A549 cell line. Experiments were performed in triplicates, and statistical analysis was performed via *t*-test (*n* = 3) (set of experiments = 3). (**B**) Efficacy of gemcitabine when administered i.p. at 120 mg/kg for 8 times in 21 days (*n* = 6) and of OralGem when administered per os daily at 6 mg/kg for 21 days (*n* = 9) in Nonobese diabetic/severe combined immunodeficiency (NOD/SCID) mice xenografted with the non-small cell lung cancer (NSCLC) A549 cell line. The control group (*n* = 7) received per os daily 0.5% CMC for 21 days. (**C**) Representative photos of histologic sections of xenograft tissue harvested from the lungs on the day of sacrifice (day 21). Brown color depicts vascular endothelial growth factor A (VEGFA) or thrombospondin-1 (TSP-1) expression. The nuclei staining is depicted in blue (hematoxylin). Representative × 10 fields are shown. Scale bar: 0.11 mm. Statistical analysis was performed by Student’s *t*-test; * *p* < 0.05, ** *p* < 0.01.

We sought to compare the anticancer efficacy achieved upon MTD and MTR chemotherapy, aiming to identify advantageous effects of MTR OralGem chemotherapy. The A549 cell line was engrafted subcutaneously to NOD/SCID immunocompromised mice, being responsive to both gemcitabine and OralGem half maximal inhibitory concentration (IC_50_) = 20.4 ± 5.1 nM and IC_50_ = 7.9 ± 1.6 μM, respectively) (Figure 2A). By the end of the experiment, the MTD group had received a total dose of 960 mg/kg, whereas the MTR group had received a total of 126 mg/kg (7-fold lower). Remarkably, MTR OralGem administration showed superior efficacy to the MTD group (Figure 2B). Based on our pharmacokinetic analysis (Figure 1A–C), following i.p. administration of gemcitabine at 120 mg/kg, an extremely high concentration of circulating gemcitabine (Cmax = 285 μΜ) was observed. Similarly, upon the per os administration of OralGem at 6 mg/kg, a Cmax of 0.37 μΜ was calculated, suggesting that upon the selected dosing schemes for efficacy studies, concentrations well over the calculated IC_50s_ could be achieved.

As VEGFA and TSP-1, key regulators of angiogenesis were previously shown to be altered upon MTR chemotherapy [16,17], we next investigated their expression in A549 subcutaneous tumors. Interestingly, our results showed that MTR chemotherapy induced a marked restriction in angiogenesis as evidenced by a decrease in VEGFA levels, concomitant with an increase in TSP-1 expression, compared to the control and MTD groups (Figure 2C). Altogether, these data provide evidence that MTR OralGem dosing exhibits increased tumor shrinkage and inhibits neoangiogenesis in A549 xenografted mice at markedly lower and nontoxic doses compared to MTD administration.

### 2.4. MTR OralGem-Treated Mice Exhibit Decreased Tumor Metastasis in the Lung

**Figure 3:**
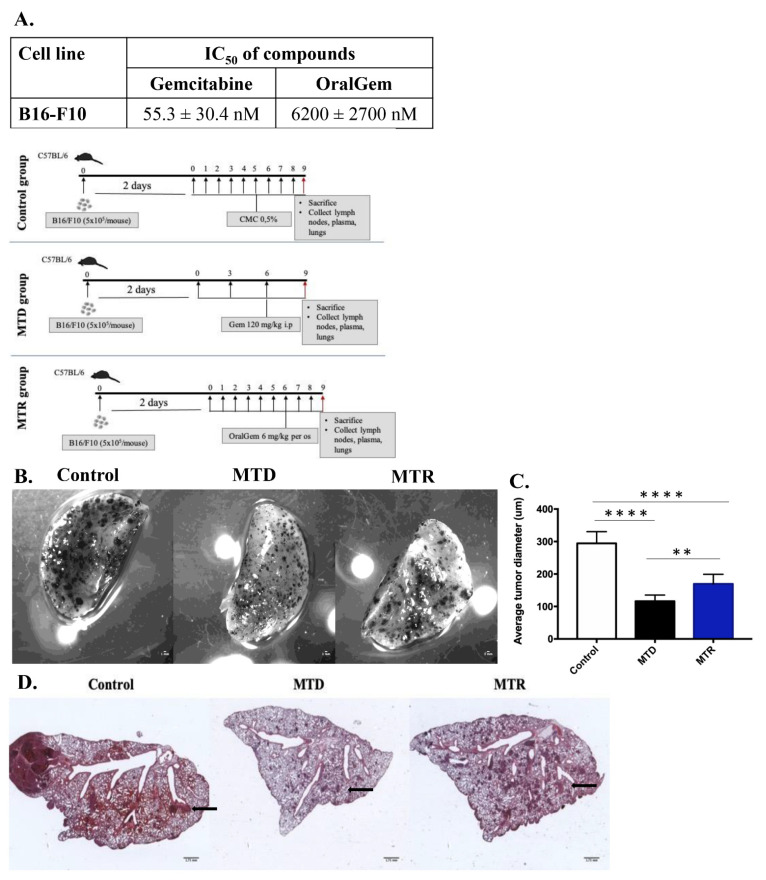
Efficacy evaluation of OralGem’s MTR daily administration in a mouse model of lung metastasis. (**A**) IC_50_ values of gemcitabine and OralGem on B16-F10 cell line. Experiments were performed in triplicates, and statistical analysis was performed via *t*-test *(n* = 3). (**B**) The efficacy of MTD and MTR chemotherapy was evaluated stereoscopically. Μale C57BL/6 mice were injected via the tail vein with B16-F10 cells (5 x10^5^ cells). Two days after cell inoculation, mice were divided randomly into 3 groups. The MTD group (*n* = 7) received i.p. gemcitabine (120 mg/kg) for 3 times in a period of 9 days. The MTR group (*n* = 7) received per os OralGem (6 mg/kg) 8 times in a period of 9 days. The control group (*n* = 8) received per os 0.5% CMC for 8 times in a period of 9 days (set of experiments = 2). (**C**) Average tumor diameter is shown. Data are mean ± SD. Statistical analysis was performed by Student’s *t*-test; ** *p* < 0.01 and **** *p* < 0.0001. (**D**) Hematoxylin and eosin (H&E) staining of the big lung lobe as indicated (1× magnification stereoscope).

As MTR chemotherapy contributes to metastasis inhibition [21,22], we decided to pursue its effects on a clinically relevant mouse model. To generate lung metastasis, we utilized the murine melanoma B16-F10 cell line and intravenously injected 5 × 10^5^ cells per mouse to C57BL/6 mice. This cell line’s IC_50_ values were 55.3 ± 30.4 nM and IC_50_ = 6.2 ± 2.7 μM for gemcitabine and OralGem, respectively (Figure 3A). Two days following cancer cell inoculation, animals were divided into three groups (control, MTD and MTR) and compound administration was initiated (Supplementary Methods S.M.6, exp. Protocol, Figure 3B). To evaluate the efficacy of the chemotherapeutic schemes, lungs were excised, and the tumors’ diameter was assessed. The control group developed tumors with an average diameter of 295 ± 36 μm, while the tumor diameters in the MTD group (116 ± 19 μm) and the MTR group (170 ± 9 μm) were significantly decreased (Figure 3C), suggesting that both MTD and MTR treatments were efficacious. Further validation of chemotherapy treatments’ efficacy through microscopic evaluation of H&E-stained lung sections confirmed the aforementioned findings (Figure 3D).

**Figure 4:**
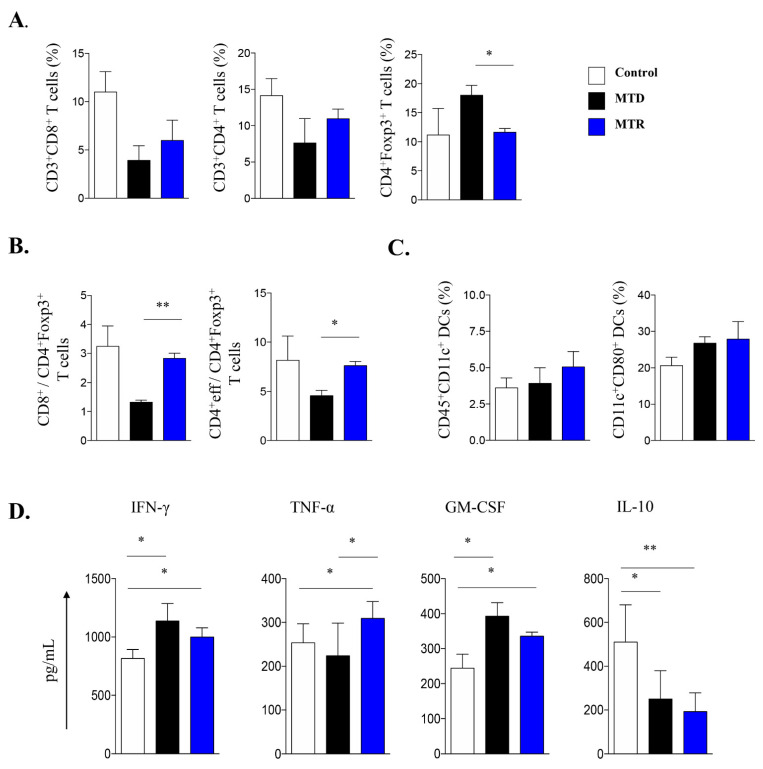
Immune cell composition in the tumor microenvironment (TME) upon MTD and MTR administration in a metastatic mouse model. (**A**) Cumulative data shown as mean ± SD depict the percentages of CD3^+^CD8^+^ cells, CD3^+^CD4^+^ cells and CD4^+^Foxp3^+^ Treg cells infiltrating lung tumors isolated by control-, MTD- or MTR-treated mice as in Figure 3B. (**B**) CD8^+^ Teff/CD4^+^Foxp3^+^ Treg and of CD4^+^Teff/Treg cell ratios in lung tumors are shown as indicated. (**C**) Cumulative data, shown as mean ± SD, depict the percentages of CD45^+^CD11c^+^ DCs and CD11c^+^CD80^+^ DCs, gated on CD45^+^ cells in lung tumors. Data are mean ± SD from *n* = 6–8 mice/group from 2 independent in vivo experiments. (**D**) IFN-γ, TNF-α, GM-CSF, and IL-10 cytokine release in cell culture supernatants of lung DLN cells re-stimulated ex vivo with antibodies against CD3/CD28. Data are mean ± SD of triplicate wells. Statistical analysis was performed by Student’s *t*-test; * *p* < 0.05 and ** *p* < 0.01.

In view of the efficacy of MTR-OralGem administration on lung tumor control in the metastatic cancer model, we next investigated immune cell composition in the TME. We observed a trend toward an increase in CD3^+^ CD4^+^ and CD3^+^CD8^+^ T effector cells infiltrating lung tumors in MTR-OralGem, compared to MTD-Gem -treated mice (Figure 4A). In contrast, Treg cell (defined as CD3^+^CD4^+^ Foxp3^+^) frequencies were significantly lower in MTR-OralGem compared to MTD-Gem -treated mice (Figure 3A,B), resulting in higher CD4^+^Teff/Treg and CD8^+^/Treg ratios upon MTR-OralGem administration (Figure 3A,B). In line with previous studies showing enhanced DC maturation upon MTR chemotherapy [23], we detected increased percentages of CD45^+^CD11c^+^CD80^+^ dendritic cells (DCs) infiltrating lung tumors in MTR-OralGem-treated mice over the other groups (Figure 4C and Figure S1). Remarkably, our findings revealed that the levels of T effector cell-associated cytokines, IFN-γ, TNF-α, were increased in culture supernatants of DLN cells obtained from mice administered with MTR-OralGem upon re-stimulation ex vivo (Figure 4D). In contrast, the release of the immunosuppressive cytokine IL-10 was decreased in DLN cells from MTR-OralGem-treated mice, compared to controls (Figure 4D). Similar to our findings in DLNs, TILs isolated from mice administered with MTR-OralGem produced increased GM-CSF levels upon re-stimulation ex vivo, accompanied by decreased ΙL-10 (Figure S1).

### 2.5. MTR OralGem Treatment Is Efficacious in a Syngeneic Model of Lung Cancer

**Figure 5:**
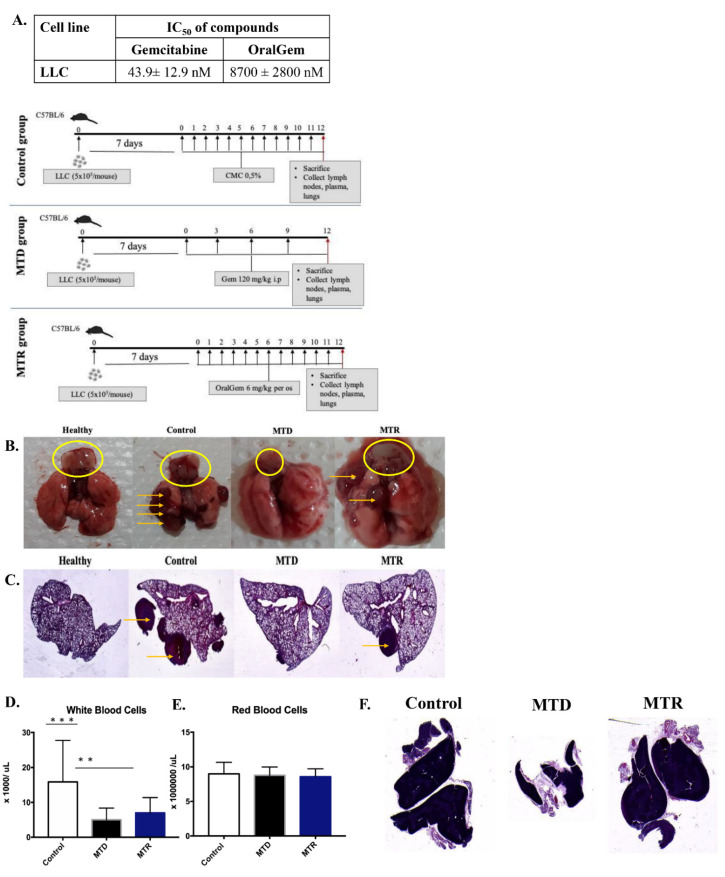
Efficacy evaluation of OralGem’s daily low dose administration in a syngeneic lung tumor model. (**A**) IC_50_ values of gemcitabine and OralGem on the Lewis lung carcinoma (LLC-OVA) cell line. Experiments were performed in triplicates, and statistical analysis was performed via *t*-test (*n* = 3). (**B**,**C**) Lungs bearing LLC-OVA tumors were resected from control, MTD, MTR-treated mice, in which LLC-OVA cells (5 × 10^5^ cells) were injected via the tail vein. Seven days after cell inoculation, mice were divided randomly into 3 groups. Lungs were evaluated macroscopically (**A**), whereas the big lung lobe was studied stereoscopically (**B**) (1x magnification) in H&E stained sections; control group (*n* = 6–8) that received no treatment, MTD group (*n* = 6–8) treated with gemcitabine 120 mg/kg for 4 times in 12 days, MTR group (*n* = 6–8) treated with OralGem 6 mg/kg for 11 days daily, are shown. The yellow arrows show the formed tumors, and the yellow circle the thymuses. Macroscopic evaluation of thymus toxicity was also performed. (**D**) Whole blood was collected for analysis of WBCs and (**E**) RBCs numbers. Data are mean ± SD. Statistical analysis was performed by Student’s *t*-test; ** *p* < 0.01 and *** *p* < 0.001. (**F**) Representative H&E staining of the thymuses, as indicated (1x magnification in the stereoscope). Data obtained by five [5] experiments.

The therapeutic benefit of MTR OralGem in a syngeneic mouse model of lung cancer was next assessed following daily administration (exp. Protocol, Figure 5B). To this end, we employed the well-established transplantable model of lung cancer via the inoculation of the Lewis lung carcinoma (LLC-OVA) cell line (5 × 10^5^ cells per mouse) in C57BL/6 mice. This cell line’s IC_50_ values were 43.9 ± 12.9 nM and IC_50_ = 8.7 ± 2.8 μM for gemcitabine and OralGem, respectively (Figure 5A). The ventilation capacity of LLC-OVA lung tumor-bearing mice was initially assessed to evaluate the severity and correspondence of the lung model [24]. As expected, the acquired measurements, such as compliance, dynamic compliance, airway resistance and central airway resistance, showed burdened ventilation capability in mice bearing LLC-OVA tumors compared to healthy mice (Figure S2).

Seven days after LLC-OVA cell inoculation, when the primary lung tumors were established, treatments with MTD gemcitabine or MTR OralGem were initiated (Supplementary Methods S.M.6). Lungs were observed macroscopically, and tumor progression was evaluated in all groups and compared to a healthy lung. Τhe MTR group had fewer tumors compared to the control group, while the MTD group exhibited the most efficacious response. Notably, MTR chemotherapy, although it generated lower circulating drug doses, exhibited considerable efficacy against lung tumors (Figure 5Β,C).

### 2.6. MTR OralGem Chemotherapy Is Characterized by Reduced Toxicity in the Syngeneic Lung Cancer Model

To test the toxicity of the two types of chemotherapy, WBCs and RBCs numbers were assessed in whole blood. A reduction in the numbers of WBCs was detected, whereas the numbers of RBCs remained the same in MTD and MTR -treated mice (Figure 5D,E). Still, MTD chemotherapy induced a slightly higher WBC reduction.

Remarkably, macroscopic and microscopic evaluations revealed that MTD gemcitabine-treated mice exhibited marked thymus toxicity, as evidenced by near-complete thymus ablation, whereas the size of the thymus remained intact in mice belonging to the MTR group and was similar to that of the control group (Figure 5F).

### 2.7. Treatment with MTR OralGem Chemotherapy Enhances Antitumor Effector Responses and Reduces Infiltration of CD4^+^Foxp3^+^ Treg Cells in the Syngeneic Mouse Lung Cancer Model

**Figure 6:**
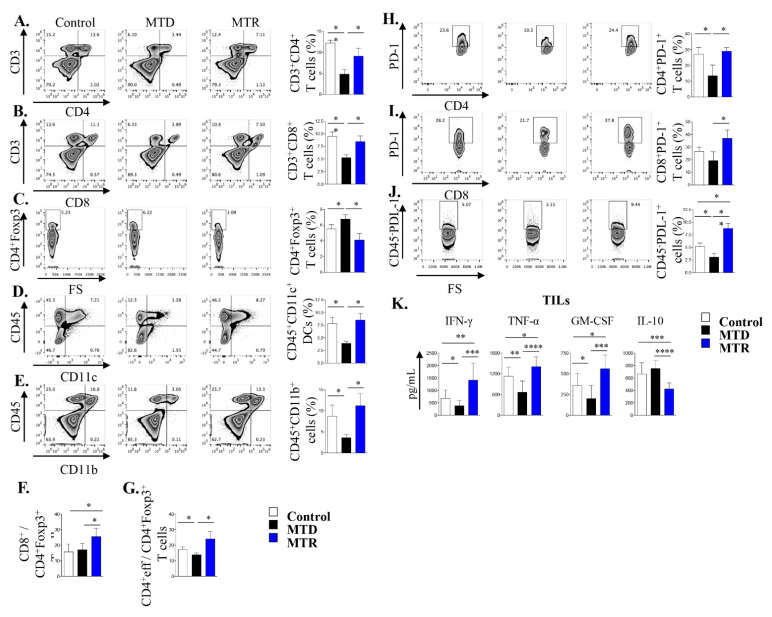
Immune cell composition in the tumor microenvironment upon MTD and MTR administration in a syngeneic lung tumor mouse model. A. Representative FACS plots showing the percentages of CD3^+^CD4^+^ T cells (**A**), CD3^+^CD8^+^ T cells (**B**), CD4^+^Foxp3^+^ Treg cells (**C**), CD45^+^CD11c^+^ DCs (**D**) and CD45^+^CD11b^+^ myeloid cells (**E**) among lung TILs isolated from LLC-OVA inoculated control-, MTD- and MTR-treated mice. Cumulative data, shown as mean ± SD, are depicted on the right. (**F**) The ratios of CD8^+^/CD4^+^Foxp3^+^ T cells and CD4^+^eff/CD4^+^Foxp3^+^ T cells (**G**) are depicted. (**H**) The percentages of CD4^+^PD-1^+^ T cells and CD8^+^PD-1^+^ T cells (**I**) are shown. (**J**) The percentages of CD45^−^PD-L-1^+^ cells are shown. Data for A-J are pooled from *n* = 6–8 mice/group from 3 independent experiments. (**K**) IFN-γ, TNF-α, Granulocyte-macrophage colony-stimulating factor (GM-CSF) and IL-10 levels in culture supernatants of TILs re-stimulated ex vivo with Ova are shown. Data are mean ± SD of triplicate wells. Statistical analysis was performed by Student’s *t*-test; * *p* < 0.05, ** *p* < 0.01, *** *p* < 0.001 and **** *p* < 0.0001.

In the next set of experiments, we explored the immunological mechanisms underlying the effects of MTR-OralGem and MTD-Gem administration, focusing on immunophenotypic analyses of distinct immune cell subsets infiltrating lung tumors. Our findings revealed that MTD-Gem chemotherapy nearly abrogated the infiltration of CD3^+^CD4^+^ and CD3^+^CD8^+^ T cells in lung tumors, than the control group (Figure 6A,B). Decreased T cell recruitment may be at least partly due to reduced thymic mass (as depicted in Figure 5F and Figure 6A,B) and is consistent with previous studies showing that high dose MTD chemotherapy can exert toxic effects not only on cancer but also on immune cells [25,26]. In contrast, MTR-OralGem administration increased the frequencies of tumor-infiltrating CD3^+^CD4^+^ and CD3^+^CD8^+^ T cells compared to MTD-Gem-treated mice, and T effector cells reached levels similar to those of the control group (Figure 6A,B). In addition, the percentages of CD45^+^CD11b^+^ myeloid cells and CD45^+^CD11c^+^ DCs in the lung TME were increased in MTR-OralGem, compared to MTD-Gem-treated mice (Figure 6D,E). In sharp contrast, MTR-OralGem treatment resulted in decreased percentages of tumor-infiltrating Treg cells compared to vehicle-treated tumors, while MTD-Gem administration increased Treg cell frequencies (Figure 6C). Together with the significant increase in tumor-infiltrating CD3^+^CD8^+^ and CD3^+^CD4^+^ T effectors, this resulted in enhanced CD4^+^Teff/Treg and CD8^+^/Treg ratios in MTR-OralGem-treated mice (Figure 6F,G).

The levels of the PD-1 inhibitory receptor on tumor-infiltrating T cells, along with those of its ligand, PD-L-1, on tumor and infiltrating myeloid cells are associated with responsiveness to ICI immunotherapies in NSCLC patients [27]. Notably, we observed that MTR-OralGem administration markedly increased the percentages of tumor-infiltrating CD3^+^CD8^+^PD-1^+^ and CD3^+^CD4^+^PD-1^+^ T cells, compared to MTD-Gem treatment (Figure 6H,I). Moreover, PD-L-1 expression was upregulated in CD45^−^ tumor cells and in CD45^+^CD11c^+^ DCs in MTR-OralGem-treated mice (Figure S3), pointing to effects of oral gemcitabine administration on PD1-PDL1 interactions.

Building on the aforementioned findings, we next investigated T effector responses in the lung TME. To address this, we isolated TILs and examined their responses upon re-stimulation with OVA ex vivo. The levels of IFN-γ and TNF-α were markedly decreased in TILs isolated from mice treated with MTD-Gem, compared to the control group, emphasizing the notion that high dose gemcitabine exerts immunosuppressive effects (Figure 6K). Remarkably, MTR-OralGem treatment significantly increased both IFN-γ and TNF-α release by TILs, compared to MTD-Gem and the control groups (Figure 6K). In contrast, IL-10 levels were decreased (Figure 6K). Finally, the levels of GM-CSF, a cytokine essential for myeloid cell maturation and recruitment, were elevated in culture supernatants of TILs obtained from mice administered with MTR-OralGem (Figure 6K). Similar findings were obtained in DLN cells upon re-stimulation with OVA ex vivo (Figure S3).

Overall, these data suggest that MTR-OralGem treatment yields robust immunostimulatory effects in the lung TME, accompanied by an upregulation of PD-1/PD-L-1 interactions between T effectors and tumor cells.

### 2.8. Co-Administration of MTR OralGem with Anti-PD1 Antibody Exhibits Increased Efficacy in the Syngeneic Lung Cancer Model

To explore whether co-administration of MTR OralGem with an immune checkpoint inhibitor leads to superior efficacy with fewer toxicities, OralGem was co-administered with an anti-PD1 blocking antibody. This choice was based on the increased expression of PD-1 in T-cell populations and of PD-L-1 in lung tumor cells upon MTR chemotherapy (Figure 6J).

**Figure 7:**
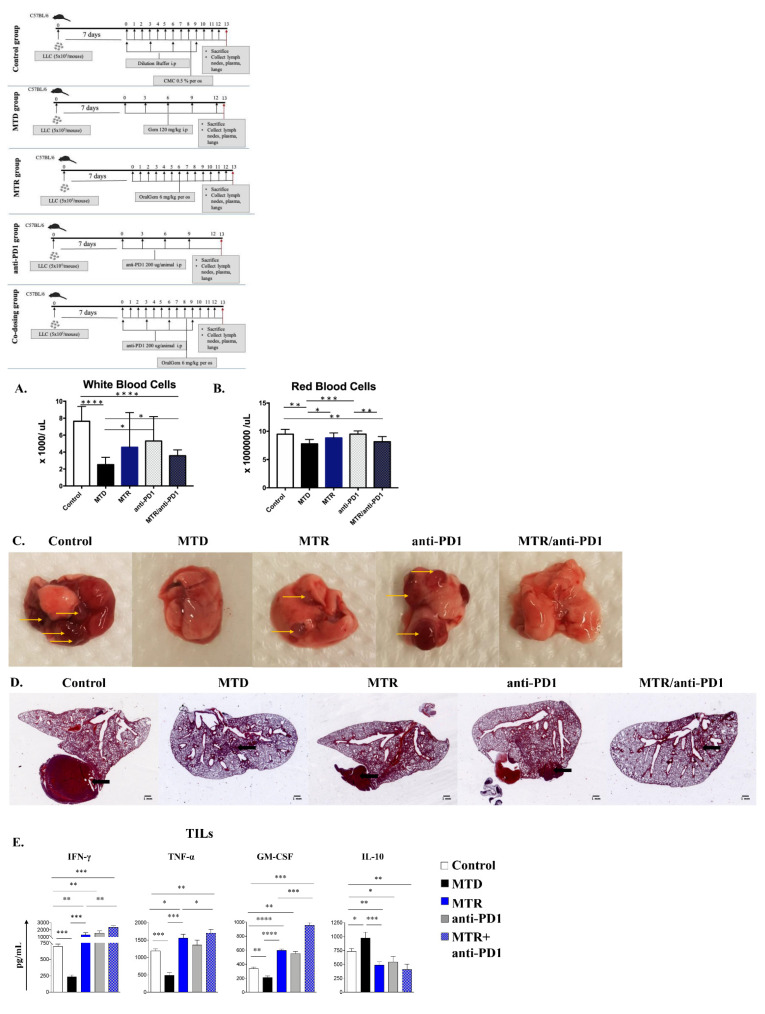
Evaluation of efficacy, toxicity and immune stimulation upon co-administration of OralGem with anti-PD1. (**A**,**B**) Whole blood was collected for analysis of WBCs and (**B**) RBCs. (**C**,**D**) Lungs bearing LLC-OVA tumors resected from control (*n* = 8), MTD (*n* = 7), MTR (*n* = 8), anti-PD1 (*n* = 8), MTR/anti-PD1-treated mice (*n* = 8) are shown (set of experiments = 1). Lungs were observed macroscopically (**C**), whereas the big lung lobe was studied stereoscopically (**D**) (1x magnification), following H&E staining. (**E**) IFN-γ, TNF-α, GM-CSF, and IL-10 levels in cell culture supernatants of TILs stimulated ex vivo with Ova are shown. Data are mean ± SD of triplicate wells from one independent experiment. Statistical analysis was performed by Student’s *t*-test; * *p* < 0.05, ** *p* < 0.01, *** *p* < 0.001 and **** *p* < 0.0001.

We used a suboptimal dose of anti-PD1 to allow assessment of possible synergy with MTR-OralGem treatment. Briefly, cohorts of LLC-OVA tumor-bearing mice were administered monotherapies consisting of anti-PD-1 antibody, MTR-OralGem, MTD-Gem or anti-PD-1 and MTR-OralGem combination therapy (Supplementary Methods S.M.6, experimental protocol Figure 7A). Initially, developing blood toxicity upon administration of distinct types of chemotherapy was evaluated. The MTD and MTR/anti-PD1 treatments induced decreased WBC numbers, compared to the control group, with MTD chemotherapy leading to a more severe WBC reduction (Figure 7A,B). MTD and MTR/anti-PD1 treatments led to a reduction in RBCs numbers compared to the other groups (Figure 7A,B). Overall, co-administration of MTR OralGem with anti-PD1 caused milder leukopenia toxicity compared to MTD treatment (Figure 7A,B).

Concerning antitumor efficacy, the group of mice that received combination therapy showed the highest efficacy compared to the other groups studied (Figure 7C,D), highlighting the synergistic effect of ICI and MTR co-administration on the reduction of lung tumor burden.

### 2.9. Increased Immunostimulatory Potential upon MTR OralGem and Anti-PD1 Co-Administration in the Syngeneic Lung Tumor Model

The aforementioned findings showing robust antitumor T cell responses upon MTR-OralGem administration prompted us to investigate the effect of combining MTR-OralGem with the anti-PD-1 blockade on antitumor immunity.

Both MTR-OralGem and anti-PD1 antibody monotherapies induced an increase in IFN-γ, TNF-α and GM-CSF release in culture supernatants following re-stimulation of TILs with OVA ex vivo (Figure 7G), compared to the control and MTD groups. Increased T effector functions were accompanied by decreased IL-10 release (Figure 7G). Like our previous findings, MTD-Gem administration decreased IFN-γ, TNF-α and GM-CSF production, concomitant with elevated IL-10, emphasizing its immunosuppressive effects (Figure 7G). Remarkably, combination therapy of MTR-OralGem and anti-PD1 antibody resulted in a synergistic enhancement of T effector responses in the lung TME as reflected by significantly increased effector cytokines in culture supernatants (Figure 7G). In contrast, IL-10 levels were decreased in T cell cultures (Figure 7G).

In aggregate, these findings highlight the potential of combining MTR-OralGem chemotherapy with immune checkpoint blockade as an effective approach for lung tumor regression associated with induction of robust antitumor immunity.

## 3. Discussion

Although conventional chemotherapy seems to be an efficacious treatment for patients with NSCLC due to its capacity to kill cancer cells, acquisition of tumor resistance and relapse is often observed. Importantly, most patients with advanced NSCLC cannot tolerate the toxic effects of MTD chemotherapy, leading to poor prognosis. Hence, clinicians have turned to complementary immunotherapy treatments. However, the success of immunotherapy is linked to the mutational load in patients’ cancer cells [28], a fact that ultimately leads to limited efficacy and even tumor progression. In search of alternative strategies, we investigated the merits of MTR treatment. Our studies revealed that the MTR administration of OralGem in an NSCLC xenografted mouse model restrained neoangiogenesis by altering two main mechanisms: increasing the expression of the anti-angiogenic factor TSP-1 and decreasing the pro-angiogenic factor VEGFA. Importantly, the robust effects of MTR OralGem administration on limiting neoangiogenesis led to effective tumor restriction. In agreement, previous studies have shown that the MTR schedule of several drugs, such as vinorelbine, cyclophosphamide, bevacizumab, gemcitabine and temozolomide in solid tumors and in multiple myeloma improves therapeutic outcomes by directing the drug towards the tumor vasculature or by increasing the expression of TSP-1 and limiting angiogenesis and vasculogenesis at the tumor [29].

An intriguing finding of our studies was that MTR OralGem treatment enhanced antitumor immunity in a syngeneic lung cancer model by decreasing the percentages of CD4^+^Foxp3^+^ Treg cells and increasing CD3^+^CD4^+^ and CD3^+^CD8^+^ T effector cell infiltration in the TME. In sharp contrast, MTD gemcitabine chemotherapy exhibited immunosuppressive functions, as evidenced by increased Treg cell infiltration and decreased T effector responses. Consistently, secretion of effector cytokines, INF-γ, TNF-α and GM-CSF by TILs were also elevated upon low-dose MTR OralGem, while IL-10 production was decreased, emphasizing its enhanced immunostimulatory potential, compared to MTD treatment. In line with our findings, MTR administration of cyclophosphamide in end-stage cancer patients reduced Treg cells while enhancing T and NK cell effector functions [30]. Moreover, other studies have demonstrated that MTD chemotherapy increases the recruitment of pro-angiogenic endothelial cells and myeloid-derived suppressor cells (MDSCs) to the TME, resulting in accelerated tumor progression [31]. The systemic release of proinflammatory and pro-fibrotic cytokines and growth factors following MTD chemotherapy contributes to metastasis, negating its tumoricidal effects [32]. Notably, our findings in the metastatic cancer model demonstrated that enhanced T effector responses in MTR OralGem-treated mice were associated with increased antigen-presenting functions on tumor-infiltrating DCs, as evidenced by increased percentages of CD45^+^CD11c^+^ cells expressing the costimulatory molecule, CD80. Importantly, MTR OralGem chemotherapy decreased lung tumor burden in the metastatic model. In support, previous studies have shown that MTR chemotherapy induces immunogenic cancer cell death, which releases danger signals that promote DC maturation and presentation of tumor antigens to CD4^+^ T cells, polarizing them towards antitumor IFN-γ-producing T helper 1 (Th1) responses [33]. In addition, MTR capecitabine reduced the frequencies of pro-tumorigenic MDSCs in glioblastoma patients [34] while MTR gemcitabine treatment decreased MDSCs in mice bearing orthotopic pancreatic tumors [22]. Metronomic cyclophosphamide also affected innate immune responses in a glioma model as evidenced by enhanced accumulation of NKs, DCs and macrophages in the TME [23].

Increased PD-1 expression in T cells and PD-L1 in tumor cells are predictive of enhanced efficacy of ICI immunotherapies [35]. We found that MTR OralGem administration enhanced PD-1 expression in lung tumor-infiltrating CD4^+^ and CD8^+^ T effectors and boosted PD-L1 levels in DC and tumor cells, sensitizing the TME to anti-PD-1 immunotherapy in the syngeneic lung cancer model. Of clinical relevance, we demonstrated that MTR OralGem chemotherapy was associated with decreased blood and thymus toxicities, compared to MTD treatment. This finding was ab initio one of the study’s goals, and highlighted MTR OralGem as a well-tolerated and safe therapy. Altogether, these data propose that our therapeutic protocol involving MTR OralGem administrations is accompanied by extensive immune reprogramming of the TME and may endow significant overall survival benefits.

Based on MTR OralGem efficacy’s immune-priming effects, we administered adjunct anti-PD-1 immunotherapy in the syngeneic lung cancer model to explore the efficacy and the generation of durable immune responses. As expected, this combination therapy led to the successful elimination of lung tumors, accompanied by increased antitumor T effector responses and cytokine release in the TME, features that surpassed MTD chemotherapy effects. Notably, considering that combination therapy augmented the efficacy of anti-PD-1 monotherapy in lung tumor regression associated with decreased blood toxicity, our findings uncover this regimen as a highly effective and advantageous treatment modality for NSCLC. In line with our findings, a recent study demonstrated that MTR paclitaxel improved the efficacy of anti-PD-1 immunotherapy in triple-negative breast cancer (TNBC) mouse model, accompanied by enhanced immunostimulatory functions [36]. It should be noted that our initial focus was the study of NSCLC. However, as the project evolved, the animal models used for this study included a murine melanoma and a lung cell line, which are not exclusively representative of NSCLC. Thus, we believe that our research can be broadened to the therapy of lung cancer in general and its metastases. Τhe TONIC trial also showed that MTR cisplatin and doxorubicin administration enhanced the clinical benefit of anti-PD-1 immunotherapy in TNBC patients [37].

When it comes to clinical trials, the effects of MTR OralGem chemotherapy have not been investigated. The most commonly-used drugs administered metronomically are cyclophosphamide, methotrexate, capecitabine, vinorelbine and etoposide [34], all given orally to ensure patient compliance. Notably, a recent relevant study to our work showed that in a murine model of pancreatic ductular adenocarcinoma (PDCA) liver metastasis, mice that received a combination of gemcitabine hydrochloride (Gem) plus anti-PD1 antibody exhibited fewer metastatic foci in the liver and prolonged overall survival compared to mice treated with Gem or anti-PD1 alone. Moreover, mice administered with this combinatorial therapy demonstrated enhanced intratumoral infiltration of cells of both the innate and adaptive immune system, such as CD11b+F4/80+Ly6C+Ly6G− inflammatory monocytes, CD11b+F4/80highCD206+ M1 macrophages and IFN-γ-expressing CD8a+ and CD4+ T cells with well-known proinflammatory/antitumor properties. In addition, the authors observed a significant upregulation in gene expression of several effector cytokines and chemokines related to anticancer immunity in tumor-infiltrating inflammatory cells [38]. Another interesting study revealed that in mice inoculated subcutaneously (sc) with LLC cells, administration of both gemcitabine and anti-PD1 resulted in reduced tumor volume and prolonged overall survival of recipients compared to those that received only gemcitabine or anti-PD1. This beneficial effect was associated with increased numbers of tumor-infiltrating CD4+ and CD8+ T cells concomitantly with reduced percentages of regulatory T cells. Moreover, tumor cells from mice that received the above combinatorial scheme produced significantly lower levels of the immunosuppressive cytokine TGF-beta along with heightened levels of the effector cytokines IL-12 and IFN-γ. Finally, combinatorial administration of gemcitabine and anti-PD-1 also inhibited postsurgical recurrence in the LLC mouse model [39]. However, except in a few cases, most trials (preclinical or clinical) do not include pharmacokinetic/pharmacodynamic correlation studies, leading to a lack of data required for MTR dose optimization. This gap in our knowledge suggests that there is no established MTR dose for either global or personalized treatment [40]. Still, MTR could provide a viable paradigm for health economics. The supply alone of antibodies as monotherapy costs thousands of US dollars every year, and this cost rises when other antineoplastic agents are co-administered [41,42]. Patients who undergo MTD chemotherapy spend short stays in hospitals and often need extensive hospitalization, adding an extra economic burden. In this context, the MTR oral delivery of gemcitabine represents a promising therapeutic approach that can achieve less toxicity, better efficacy, sustained systemic exposure, the flexibility of administration and optimized clinical outcome. In fact, recently, FUJIFILM Corporation and Merck and Co. announced the initiation of phase I clinical trial intending to evaluate the effect of FF-10832, a liposomal drug slowly releasing gemcitabine when co-administered with KEYTRUDA^®^ in advanced solid tumors [43].

Collectively, the present studies involving integrated pharmacokinetics, toxicity and immunological findings propose that MTR OralGem, combined with ICI immunotherapy, can be considered as a strong candidate scheme for the treatment of NSCLC, as it combines the desired efficacy with minimized arisen toxicities.

## 4. Materials and Methods

### 4.1. Drugs and Synthesis

Gemcitabine hydrochloride was purchased by Carbosynth Limited and solubilized in sterile H_2_O for in vitro evaluations and in saline for in vivo administration. OralGem was synthesized upon the reaction of gemcitabine hydrochloride with valproic acid, following a previously described procedure [16] (Supplementary Methods S.M.1). The synthesis was performed at the Department of Pharmacy of the National and Kapodistrian University of Athens. OralGem was diluted in 0.1% DMSO for in vitro assays and in 0.5% carboxymethyl cellulose (CMC) for animal studies.

### 4.2. Cell Lines

The A549 human lung epithelial carcinoma cell line (purchased by ATCC), the murine ovalbumin (OVA) expressing Lewis lung carcinoma (LLC-OVA) cell line (kind offer of Dr. Tsoumakidou Laboratory, BSRC Alexander Fleming) and the murine B16-F10 melanoma cell line (by ATCC, kind offer of Dr. Panoutsakopoulou Laboratory, BRFAA) were maintained in DMEM/F-12 medium, containing glutamine and supplemented with 10% fetal bovine serum (FBS), penicillin (100 U/mL), and streptomycin (100 mg/mL) at 37 °C and 5% CO_2_.

### 4.3. Mice

C57BL/6 and NOD/SCID (Charles River Laboratory, Wilmington, MA, USA) were maintained at the BRFAA animal facility.

### 4.4. Cell Growth Inhibition

The antiproliferative activity of gemcitabine and OralGem was evaluated by the MTT Assay, as described previously [43]. Details of the protocol are available in the Supplementary Methods S.M.2.

### 4.5. Drug Doses and Schedules and Pharmacokinetic Analysis

Gemcitabine was administered i.p. at 120 mg/kg in saline, as this dosage simulates MTD chemotherapy used in the clinic [44]. OralGem was administered orally at 6 mg/kg in 0.5% carboxymethyl cellulose (CMC), the lowest dose shown to be efficacious and not toxic when administered daily [17]. The dosing schemes followed in each experiment are described in detail in Supplementary Methods S.M.7. Blood samples were collected and prepared as described previously [43] (Supplementary Methods S.M.3). The concentrations of gemcitabine, OralGem and dFdU in blood were determined by LC–MS/MS analysis (Supplementary Methods M.S.4).

### 4.6. In Vivo Toxicity Evaluation

Naive male C57BL/6 mice were divided in 3 groups, the control (*n* = 5), the MTD (*n* = 5) and the MTR (*n* = 6) group (Supplementary Methods S.M.5). Mice were sacrificed, and whole blood was collected by cardiac puncture and analyzed in Nihon Kohden CELLTAC.

### 4.7. Tumor Models

A xenograft mouse model was used to evaluate the efficacy of MTR OralGem and its effects on angiogenesis. Briefly, male NOD/SCID mice were used, and primary xenografts were generated after the engraftment of A549 cells in both flanks of mice (10^6^ cells/flank). Treatment was initiated when tumors reached 100 mm^3^ (Supplementary Methods S.M.6). In another lung cancer model, lung tumors were developed orthotopically after the intravenous (i.v.) administration of murine LLC-OVA expressing cells (5 × 10^5^ cells/animal) in male C57BL/6 mice as described previously (Supplementary Methods S.M.6). To model metastatic disease, melanoma tumors in the lungs were generated after i.v. administration of B16-F10 melanoma cell in male C57BL/6 mice (5 × 10^5^ cells/animal) (Supplementary Methods S.M.6). Mediastinal lung-draining lymph nodes (DLNs) and tumor-infiltrating lymphocytes (TILs) were isolated from lung tumor-bearing mice and used in ex vivo stimulation assays (Supplementary Methods S.M.7) ELISA and FACS analyses (Supplementary Methods S.M.8).

### 4.8. Tissue Immunostaining for Markers of Angiogenesis

Excised tumors/lungs were fixed in 4% paraformaldehyde, paraffin-embedded, sectioned and stained with antibodies against VEGFA and TSP-1. Angiogenesis was assessed on tumors in a 10× microscope field. Images were acquired by a Leica DFC350-FX camera mounted on a Leica DMLS2 microscope.

### 4.9. Isolation of TILs

Mice were euthanized, and lung tumors were excised using sterile scissors and forceps and minced into small pieces using two single-edged razor blades. Single-cell suspensions were performed using 70 µm and 40 µm cell strainers (Corning). Cells were resuspended in 40% Percoll (Sigma-Aldrich), layered over an equal volume of 80% Percoll and centrifuged at 600× *g* for 30 min with minimum deceleration. TILs were recovered from the interphase.

### 4.10. Flow-Cytometry Analysis

TILs were stained with fluorescently labeled antibodies to CD45, CD4, CD8, CD3, CD11c, CD11b, PD1, PDL-1 and Foxp3 (eBiosciences). For Foxp3 staining, cells were permeabilized and fixed using a commercially available kit (eBioscience) according to the manufacturer’s instructions. FACS acquisition was performed with the cytometer Cytomics FC500 (Beckman Coulter), and the LSRFortessaTM cytometer (BD Biosciences) and data were analyzed using the FlowJo software. The antibodies used for the analyses were the following and were purchased by: CD45 (clone: 30-F11, Invitrogen), CD4 (clone: GK1.5,eBioscience), CD8 (clone: 53–6.7, BioLegend), CD3 (clone: 17A2, BioLegend),CD11c (clone: N418, eBioscience), CD11b (clone: M1/70, BioLegend), PD1 (clone: J43, eBioscience), PDL-1 (clone: MIH1, BD Biosciences), Foxp3 (clone: FJK-165, eBioscience).

### 4.11. Statistical Analyses

Statistical analyses and calculation of IC_50_ values were performed by GraphPad Prism software (version 7). Statistical significance was determined using the Student’s two-tailed, two-sample unpaired variance distribution *t*-test [45].

## 5. Conclusions

Upon MTR OralGem administration, alterations in the angiogenic profile around the tumor site were detected, as well as the recruitment of immune populations, limiting inflammation and boosting immune surveillance. The metronomic dosing led to decreased angiogenesis and metastatic potential of NSCLC tumors, along with enhanced immunostimulatory reprogramming in the tumor microenvironment, acting synergistically in favor of tumor restriction. The minimized blood and thymus toxicity upon metronomic treatment concomitant with the enhanced antitumor efficacy achieved when oral gemcitabine was co-administrated with immunotherapy represent key findings of the study.

Overall, our studies demonstrate that the combination of MTR OralGem with immunotherapy is highly efficacious and tolerable, illuminating it as a strong candidate therapeutic scheme for the treatment of NSCLC.

## Data Availability

The datasets supporting the conclusions of this article are included within the article and the supplementary material.

## Supplementary Materials

The following are available online at https://www.mdpi.com/article/10.3390/cancers13081901/s1. Supplementary Figures S1–S3; Supplementary Methods: S.M.1 OralGem synthesis, S.M.2. MTT Assay, S.M.3 In vivo pharmacokinetic evaluation of gemcitabine and OralGem, S.M.4 LC–MS/MS analysis for the simultaneous quantification of OralGem, gemcitabine and dFdU, S.M.5 In vivo toxicity studies, S.M.6 Animal Models, S.M.7 Ex vivo stimulation assays, S.M.8 Cytokine analysis.

## Availability of Data and Materials

The datasets supporting the conclusions of this article are included within the article.
