# Supplementary material for: Immunotherapy Combined with Metronomic Dosing: An Effective Approach for the Treatment of NSCLC
Source: Cancers (Basel). 2021 Apr 15;13(8):1901. doi: 10.3390/cancers13081901 (PMC8071233; doi:10.3390/cancers13081901)
Supplement: Supplementary file 1 [file cancers-13-01901-s001.zip › cancers-1160677-supplementary.pdf]

Supplementary Information

Supplementary Figures

S.F.1

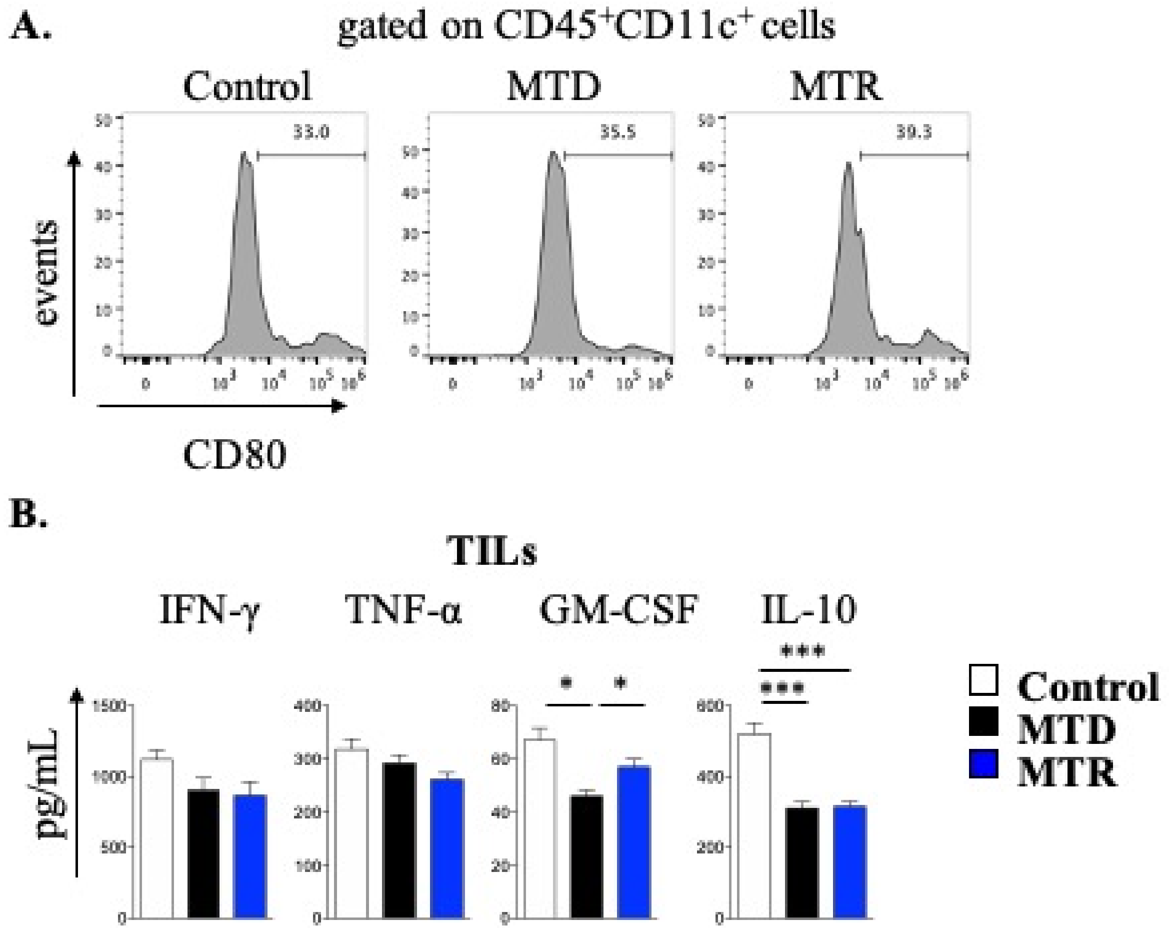

S.F.1: Male C57BL/6 mice were injected via the tail vein with B16-F10 cells (5 x10^5 cells). Two days after cell inoculation, mice were divided randomly in 3 groups. The MTD group (*n*=7) received i.p. gemcitabine (120 mg/kg) for 3 times in a period of 9 days. The MTR group (*n*=7) received per os OralGem (6 mg/kg) for 9 times in a period of 9 days. The Control group (*n*=8) received per os 0.5% CMC for 9 times in a period of 9 days. Every 3 days, the weight of the animals was examined. Experiments were terminated after 9 days by euthanizing the animals under ketamine/xylazine anesthesia. Whole blood was collected for hematological analysis, the big lung lobe was kept for H&E analysis and the rest of the lung lobes were collected for isolation of TILs and *ex vivo* proliferation assays. **A.** Expression of the CD80 costimulatory molecule on the surface of $CD45^+CD11c^+$ DCs infiltrating lung tumors in MTR-OralGem-treated mice. **B**. Tumor-infiltrating T lymphocytes isolated from mice, treated as indicated, were re-stimulated *ex vivo*. Cytokine release in culture supernatants is shown. Data are mean ± SEM of triplicate wells from 2 independent experiments. Statistical analysis was performed by Student's t test; *P < 0.05, **P < 0.01 and ***P < 0.001.

S.F.2

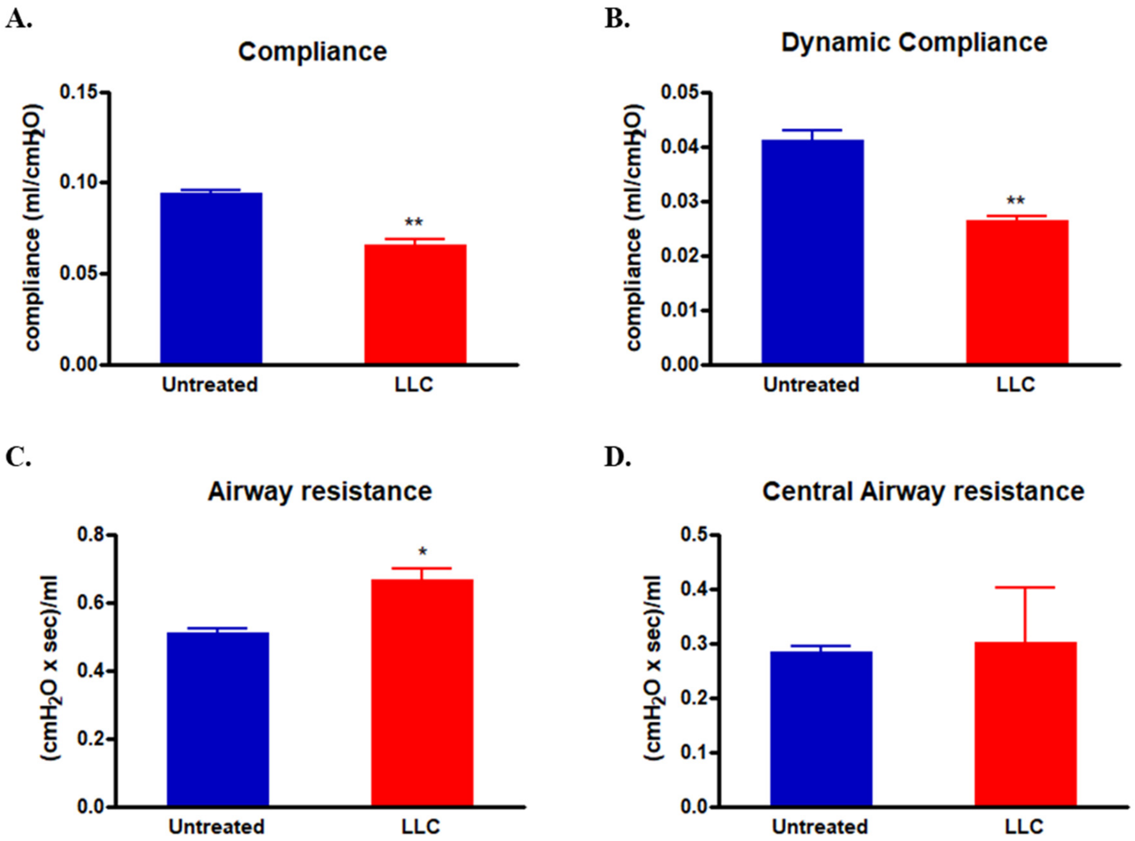

S.F.2: Lung mechanics measured in anesthetized mechanically-ventilated mice (flexiVent, SciReq, Montreal, Canada) as previously described (48). C57BL/6 mice were injected intravenously with LLC cells ($5x10^5$ cells/mouse). Twenty-seven (27) days post cell inoculation, two mice were used for the evaluation of their lung mechanics and were compared to three healthy mice. Four measurements were assessed for this evaluation. **A.** Compliance, **B**. Dynamic compliance, **C**. Airway resistance, **D**. Central airway resistance.

S.F.3

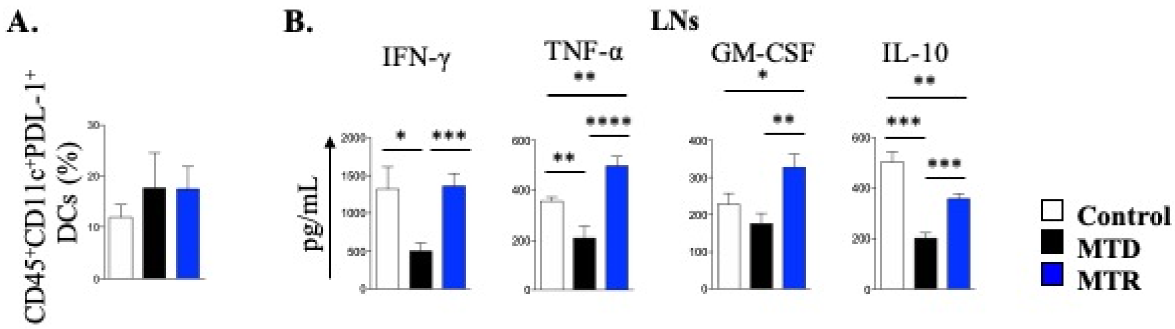

S.F.3: Male C57BL/6 mice were used, in which LLC cells ($5 x10^5$ cells) were injected via the tail vein. Seven days after cell inoculation, mice were divided randomly in 3 groups. The MTD group received i.p. gemcitabine (120 mg/kg) for 4 times in a period of 11 days. The

MTR group received per os OralGem (6 mg/kg) for 11 times in a period of 11 days. The Control group received per os 0.5% CMC for 11 times in a period of 11 days. Every 3 days, the weight of the animals was examined. Experiments were terminated after 11 days by euthanizing the animals under ketamine/xylazine anesthesia. Whole blood was collected for hematological analysis, the big lung lobe was kept for H&E analysis and the rest of the lung lobes were collected for isolation of TILs and *ex vivo* stimulation assays. **A**. Percentages of $CD45^{+}CD11c^{+}PDL\text{-}1^{+}$ DCs infiltrating lung tumors are shown. Data are mean ± SEM pooled from *n*=6-8 mice/group from 3 independent experiments. **B**. Lung-DLN cells, isolated from mice treated as indicated, were re-stimulated *ex vivo* with OVA. Cytokine release in culture supernatants is shown. Data are mean ± SEM of triplicate wells. Statistical analysis was performed by Student's t test; *P < 0.05, **P < 0.01, ***P < 0.001 and ****P < 0.0001.

Supplementary Methods

S.M.1 OralGem synthesis

S.M.1.1. General Information

^{1}H-NMR, ^{13}C-NMR and 2D (COSY, NOESY, HSQC, HMBC) NMR spectra were recorded on a Bruker Avance III 600 instrument, in deuterated dimethylsulfoxide-d_6 and were referenced to TMS (δ scale). The signals of ^{1}H and ^{13}C spectra were unambiguously assigned by using 2D NMR techniques: ^{1}H^{1}H COSY, NOESY, HSQC and HMBC. Column chromatography was performed on Merck silica gel 60 (0.040–0.063 mm). Analytical thin layer chromatography (TLC) was carried out on precoated (0.25 mm) Merck silica gel F-254 plates. Gemcitabine hydrochloride was purchased by Carbosynth Limited. The rest of the chemical reagents (N-methylmorpholine, 1-hydroxybenzotriazole hydrate, valproic acid and EDCI hydrochloride) were purchased from Acros Organics and were used without further purification. Anhydrous N,N-dimethylformamide (DMF) and dimethyl sulfoxide (DMSO) were purchased from Acros Organics in AcroSeal® bottles.

S.M.1.2. Synthesis of N-(1-((2R,4R,5R)-3,3-difluoro-4-hydroxy-5-(hydroxymethyl)tetrahydrofuran-2-yl)-2-oxo-1,2-dihydropyrimidin-4-yl)-2-propylpentanamide (OralGem, **2**).

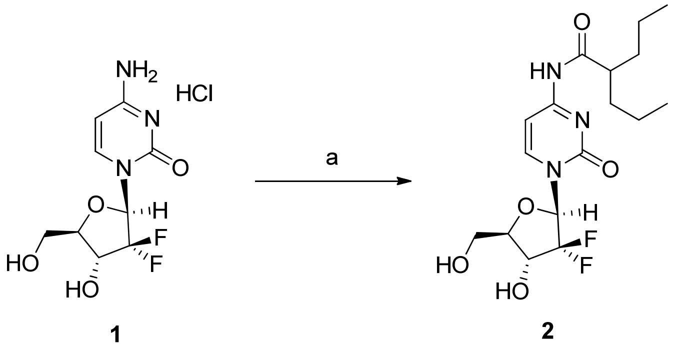

Reagents and conditions: a) NMM, HOBt hydrate, valproic acid, EDCI hydrochloride, DMF / DMSO, 55 °C, Ar, 20 h.

N-Methylmorpholine (185 μL, 1.68 mmol) was added into a suspension of gemcitabine hydrochloride (**1**, 500 mg, 1.67 mmol) in a mixture of anhydrous DMF / DMSO (1.5 mL / 0.5 mL, respectively), under argon. Then 1-hydroxybenzotriazole hydrate (260 mg, 1.70 mmol), valproic acid (293 μL, 1.87 mmol) and finally EDCI hydrochloride (416 mg, 2.17 mmol) were added and the reaction mixture was stirred at 55 °C, under argon, for 20 hours. Upon completion of the reaction, the mixture was allowed to reach room temperature and then an aqueous solution of NaCl (10% w/v, 2.5 mL) and water (1.5 mL) were added, followed by extraction with ethyl acetate (4 x 3 mL). The combined organic layers were subsequently washed with an aqueous solution of LiCl (20% w/v, 2 x 1.5 mL), a saturated aqueous solution of $NaHCO_3$ (2.5 mL) and finally with a saturated aqueous solution of NaCl (1.5 mL). The organic layer was then collected, dried over sodium sulfate and evaporated under reduced pressure to provide the crude amide **2** as a pale-yellow oil. The crude product was purified by column chromatography using a mixture of dichloromethane / methanol (from 100 / 1 up to 100 / 10, v / v) as the eluent, to provide the pure amide **2** (480 mg, yield 74%) as a white foam. ^{1}H-NMR (DMSO-d_6, 600MHz) δ 0.84 (t, 6H, J = 7.3 Hz, 2 x CH_3), 1.17-1.27 (m, 4H, 2 x $C\underline{H}_2CH_3$), 1.30-1.36 (m, 2H, $C\underline{H}_2CH_2CH_3$), 1.49-1.55 (m, 2H, $C\underline{H}_2CH_2CH_3$), 2.59-2.64 (m, 1H, $C\underline{H}(CH_2CH_2CH_3)_2$), 3.64-3.68 (m, 1H, H-5'), 3.78-3.83 (m, 1H, H-5'), 3.88-3.91 (m, 1H, H-4'), 4.16-4.24 (m, 1H, H-3'), 5.29 (t, 1H, D_2O exch., J = 5.4 Hz, OH-5'), 6.17 (t, 1H, J = 7.4 Hz, H-1'), 6.31 (d, 1H, D_2O exch., J = 6.5 Hz, OH-3'), 7.32 (d, 1H, J = 7.6 Hz, H-5), 8.24 (d, 1H, J = 7.6 Hz, H-6), 11.00 (s, 1H, D_2O exch., CONH). ^{13}C-NMR (151 MHz, DMSO-d_6) δ 13.92 (CH_3), 20.06 ($\underline{C}H_2CH_3$), 34.48 ($\underline{C}H_2CH_2CH_3$), 45.88 ($\underline{C}H(CH_2CH_2CH_3)_2$), 58.85 (C-5'), 68.32, 68.47, 68.62 (C-3'), 81.14 (C-4'), 84.01, 84.23, 84.43 (C-1'), 96.08 (C-5), 121.27, 122.98, 124.70 (C-2'), 144.89 (C-6), 154.31 (C-2), 162.93 (C-4), 177.40 (CO).

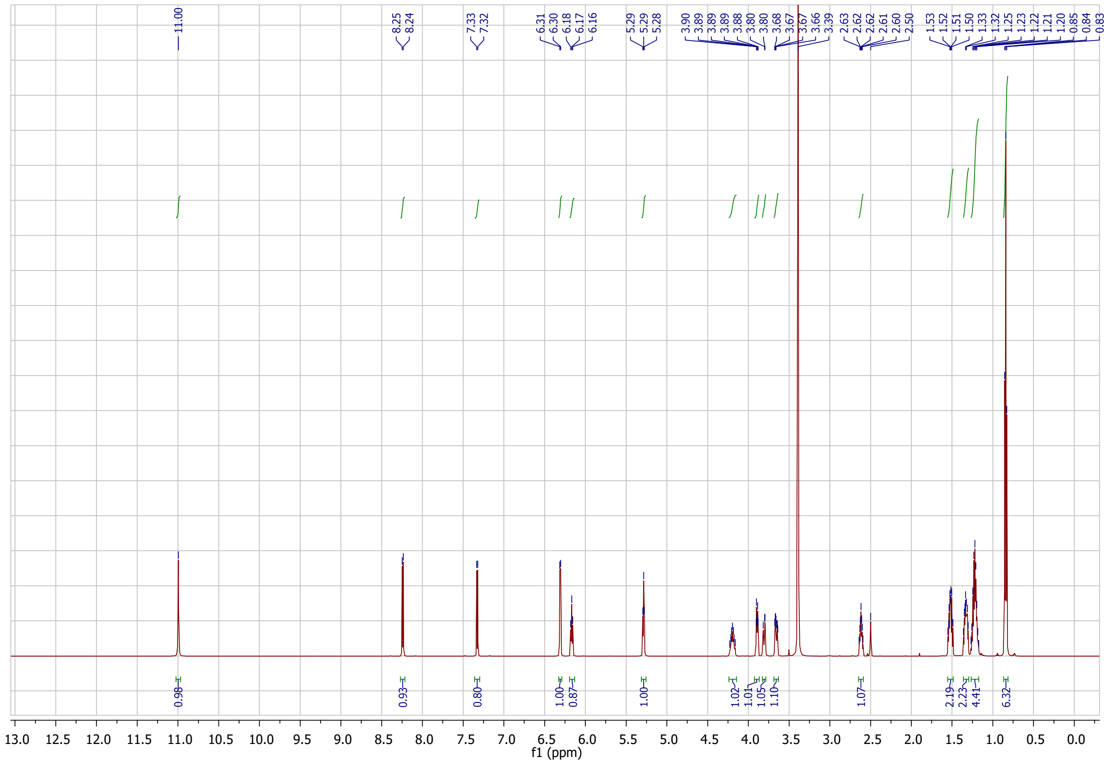

^{1}H-NMR spectrum of compound **2** in DMSO-d_6.

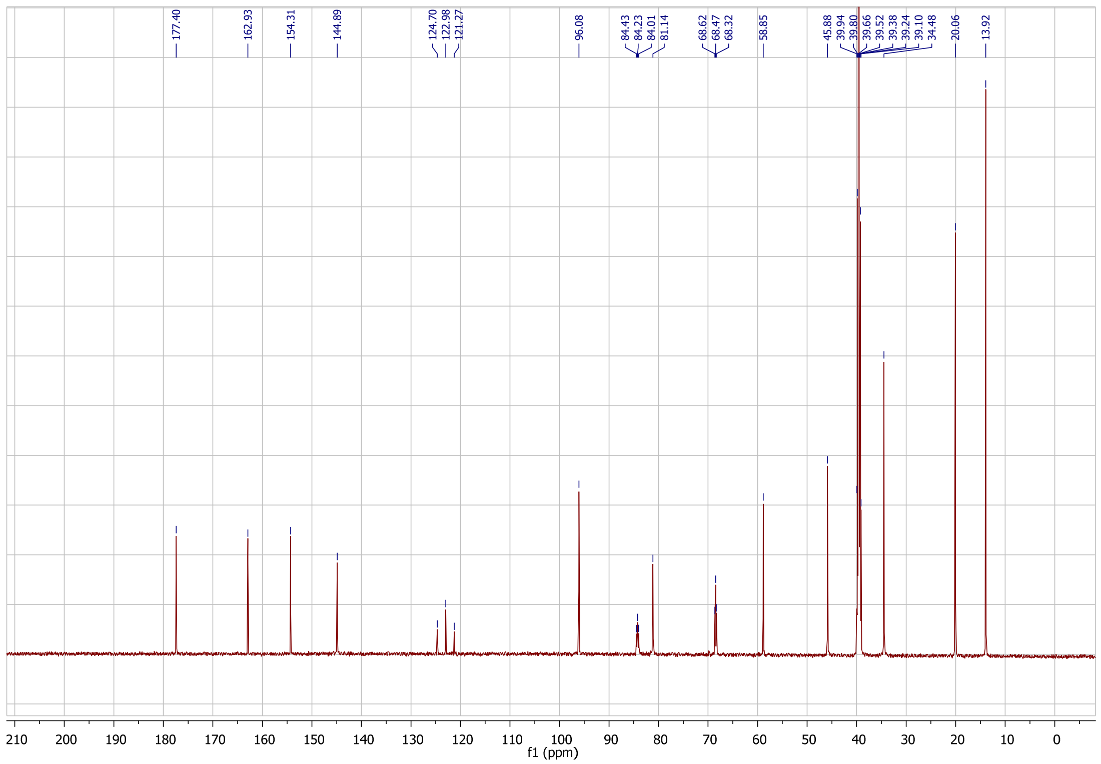

^{13}C-NMR spectrum of compound **2** in DMSO-*d*$_6$.

S.M.2 MTT Assay

A549 cells were plated at a density of 5 × 10^3 cells per well on 96-well plates. After 24 h of incubation (37 °C, 5% CO_2), the cell medium was removed, and various concentrations of gemcitabine (in saline) and OralGem (in 0.1% DMSO) were added to cells. After 72 h of drug incubation, the medium was removed and the MTT solution (0.3 mg/mL in PBS) was added to cells for 3 h, after which the MTT solution was removed and 100 µL of DMSO was added. The optical density was measured at 570 nm and a reference wavelength of 690 nm using an absorbance microplate reader (SpectraMax 190, Molecular Devices, Sunnyvale, CA, USA). Experiments were performed in triplicate.

S.M.3 *In vivo* pharmacokinetic evaluation of Gemcitabine and OralGem

For the pharmacokinetic experiments, a mouse serial tail bleeding protocol was developed using 12 C57BL/6 naïve mice (average age= 8-10 weeks with an average weight= 25 g/animal). All animals were weighed and fasted overnight before dosing. Animals were divided in 2 main groups:

Group No 1: 6 animals were administered intraperitoneally 120 mg/kg (15 mg/mL) gemcitabine in saline with a total volume of 200 µL/animal.

Group No 2: 6 animals were administered orally 6 mg/kg (0.75 mg/mL) OralGem in 0.5% CMC with a total volume of 200 µL/animal.

A serial tail bleeding protocol was used for the collection of blood samples. Blood samples (10 µL) were collected at seven time points (0.15 h, 0.5 h, 1 h, 2h, 4h, 6 h, 8 h, 24 h) in tubes containing 40 µL sodium citrate (0.1 M, pH 4.5) and stored at -80° C until the day of the analysis.

Samples were prepared for quantification by protein precipitation and evaporation. OralGem, Gemcitabine and dFdU were quantified by LC-MS/MS analysis, as described previously.

S.M.4 LC-MS/MS analysis for the simultaneous quantification of OralGem, gemcitabine and dFdU.

In order to quantify OralGem, gemcitabine and the inactive metabolite of gemcitabine, dFdU, a mass spectrometric method was developed for all three compounds as well as for capecitabine, that was used as internal standard (IS).

After infusion and characterization of the investigated compounds in the mass spectrometer, a Liquid Chromatography (LC) method was developed. The typical mass spectrometric properties for the LC-MS analysis were set as follows: Ion voltage: 5500 V, Ion Source temperature: 550 oC, Nitrogen Curtain: 20, collision activated dissociation (CAD) gas: 12 and Ion Source gases 1 and 2: 40 and 45 respectively (arbitrary units).

A HPLC method was developed for separation with mobile phases C to be consisted of 95% H_2O- 5% ACN, 2 mM ammonium acetate, 0.1% formic acid; and mobile phase B of 90% ACN- 10% H_2O, 2 mM ammonium acetate, 0.1% formic acid. The column used was: C18 column (ZORBAX Poroshell 120, 4.6 x 150mm, 5 µM, Agilent Technologies). The gradient methodology developed for this separation is presented below.

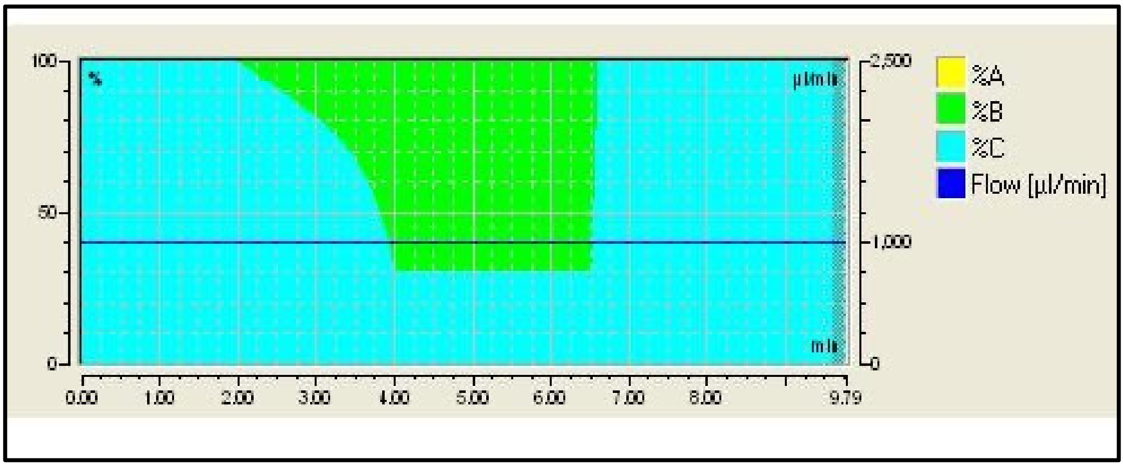

The gradient method used for the separation of OralGem, gemcitabine and its inactive metabolite, dFdU, along with capecitabine (IS). The duration of the method was 9.8 min and the steps included were: (i) 0-2 min: 100% C, (ii) 2-4 min: gradient change from 100% C to 70% B, (iii) 4-6.5 min: 70% B, (iv) 6.5-9.8 min: 100% C.

A representative chromatogram is also depicted below. The simultaneous monitoring and quantification of OralGem, gemcitabine, dFdU and Capecitabine (IS) in mouse plasma was achieved. The chosen transitions to work with were for OralGem 390.5/112.0, for gemcitabine 264.3/112.1, for dFdU 265.3/113.1 and for capecitabine 360.6/173.9 respectively. The retention time for OralGem was 5.92 min, for gemcitabine 2.19 min, for dFdU 4.39 min and for Capecitabine 5.69 min.

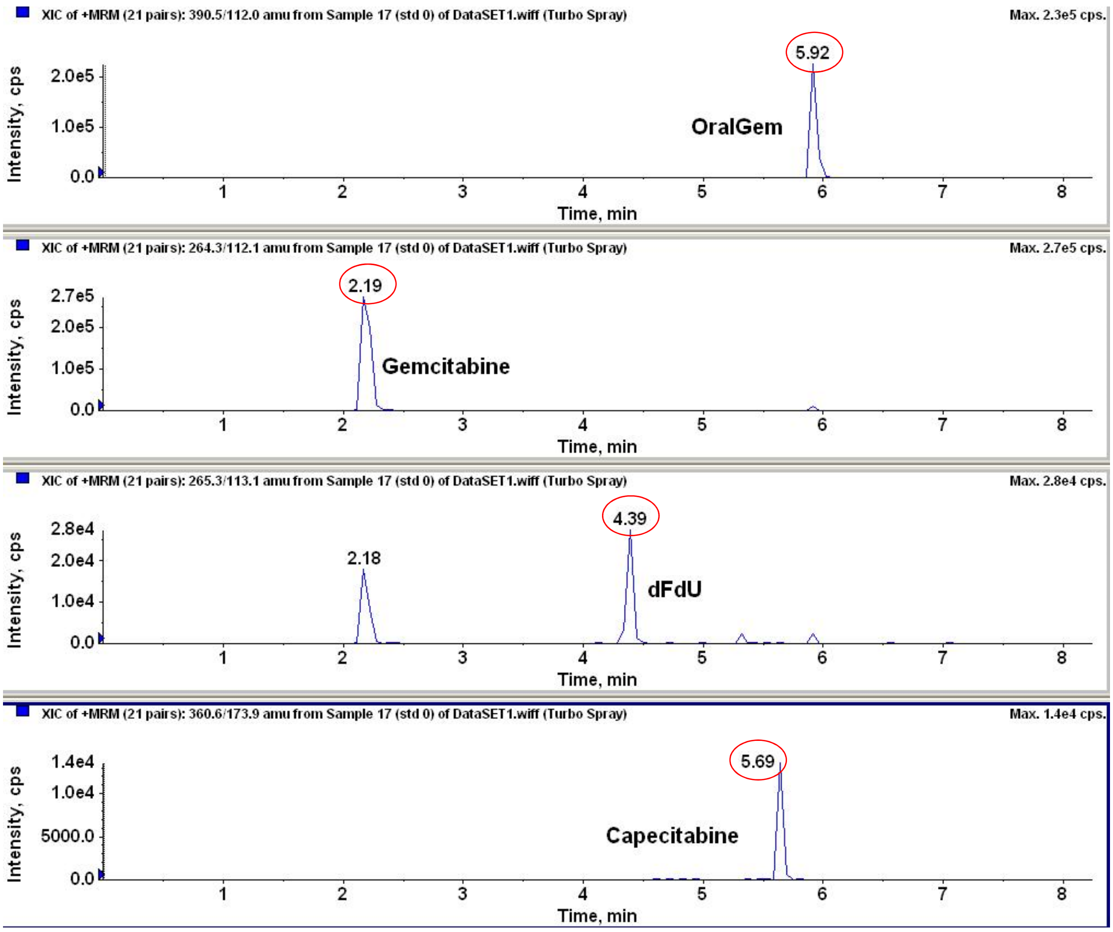

LC-MRM chromatogram showing the separation of OralGem, gemcitabine, dFdU and Capecitabine (IS).

<u>Calibration standards and quality-control samples</u>

Preparation of stock and working solutions

A set of working solutions for OralGem, gemcitabine and dFdU containing 1, 5, 10, 25, 50, 100 and 250, 500, 1000, 2500 and 5000 ng/ mL were prepared by diluting the 1 mg/mL stock solution with ACN:H_2O 1:1 into intermediate stocks. Stock solution and working solutions were stored at 4°C. Three QC samples were prepared from a 1 mg/mL stock solution with the following concentrations: 10, 100 and 1000 ng/ mL.

<u>Sample extraction</u>

Standards, QC samples and IS were left at room temperature for 20 min. Fifty µL of IS, Standards/QCs were spiked into each 50 µL aliquot of plasma (purchased by TCS Biosciences), except for the double blank, into which 50 µL of ACN:H_2O 1 : 1 was added. The solutions were then vortexed and sonicated, after which each sample was precipitated by adding 400 µL of cold ACN. Next steps included sonication, vortex, and centrifugation at 13.000 rpm for 10 min. The supernatants were then processed for evaporation (50 °C, maximum vacuum pressure, 70 min).

S.M.5 *In vivo* toxicity studies

C57BL/6 wild type mice were used for this experiment and divided in 3 main groups. In the MTD group, mice (*n*=5) were administered intraperitoneally a dose of 120 mg/kg for 8 times

in a period of 21 days, whereas in the MTR group, animals (*n*=6) were administered orally the low dose of 6 mg/kg of OralGem every day for 21 days. Finally, the Control group (*n*=5) received orally 21 times CMC 0.5%. When the protocol was completed, mice were sacrificed and whole blood was collected and analyzed for the detection of any alteration of White (WBCs) and Red Blood Cells (RBCs).

S.M.6 Animal Models

1. A549 xenografted animal model: Male NOD/SCID mice were injected with A549 cells (10^6 cells/flank). Three weeks after cell implantation, when the tumor had reached the size of 100 mm^3, mice were randomly divided in 3 groups. The MTD group received i.p. gemcitabine (120 mg/kg) for 8 times in a period of 21 days. The MTR group received per os OralGem (6 mg/kg) for 21 times in a period of 21 days. The Control group received per os 0.5% Carboxymethyl cellulose (CMC) for 21 times in a period of 21 days. Every 3 days, the weight of the animals and the tumor size were measured (by a calliper) and. Experiments were terminated after 21 days by euthanizing the animals under isoflurane anesthesia. Lung tumors were excised, fixed and prepared for immunohistochemistry.

2. LLC syngeneic animal model: Male C57BL/6 mice were used, in which Ova-expressing LLC cells (5 $x10^5$ cells) were injected via the tail vein. Seven days after cell inoculation, mice were divided randomly in 3 groups. The MTD group received i.p. gemcitabine (120 mg/kg) for 4 times in a period of 11 days. The MTR group received per os OralGem (6 mg/kg) for 11 times in a period of 11 days. The Control group received per os 0.5% CMC for 11 times in a period of 11 days. Every 3 days, the weight of the animals was examined. Experiments were terminated after 11 days by euthanizing the animals under ketamine/xylazine anesthesia. Whole blood was collected for hematological analysis, the big lung lobe was kept for H&E analysis and the rest of the lung lobes were collected for isolation of TILs and *ex vivo* stimulation assays. Mediastinal lung-DLN cells were also harvested and used in *ex vivo* stimulation assays.

In another set of experiments, mice were administered i.p. with 200 ug of anti-PD1 antibody (4 times in 13 days, one injection every 3 days), (*n*=8). In the combination therapy group (MTR/anti-PD1), animals (*n*=8) were administered orally the low dose of 6 mg/kg of OralGem every day (13 injections in 13 days), along with an i.p. injection of 200 ug anti-PD1 (4 times in 13 days) (Anti-mouse PD-1 (CD279) (CLONE: RMP1-14, by BioXCell). The parameters mentioned above were evaluated.

4. B16-F10 melanoma animal model for evaluation of lung metastasis: Male C57BL/6 mice were injected via the tail vein with B16-F10 cells (5 $x10^5$ cells). Two days after cell inoculation, mice were divided randomly in 3 groups. The MTD group (n=7) received i.p. gemcitabine (120 mg/kg) for 3 times in a period of 9 days. The MTR group (n=7) received per os OralGem (6 mg/kg) for 9 times in a period of 9 days. The Control group (n=8) received per os 0.5% CMC for 9 times in a period of 9 days. Every 3 days, the weight of the animals was examined. Experiments were terminated after 9 days by euthanizing the animals under ketamine/xylazine anesthesia. Whole blood was collected for hematological analysis, the big lung lobe was kept for H&E analysis and the rest of the lung lobes were collected for isolation of TILs and *ex vivo* proliferation assays. Mediastinal lung-DLN cells were also harvested and used in *ex vivo* stimulation assays.

In order to evaluate the efficacy of both types of treatment in this animal model, ~~we used the~~ Photoshop Software was used, and its extension called “Count Tool”. Representative photos were taken using a stereoscope and then via the Count Tool, ~~we tried to count~~ all visible tumors were counted, which were depicted as black dots. ~~Additionally, we decided to measure t~~The diameter of the tumors in the various treatments were also counted, using a ruler which converted the pixels to mm. This ruler tool let us measure distances in pixels. In detail, there is a certain scale for each magnification length of a stereoscope/microscope. We chose the proper one for the camera of the stereoscope used for this analysis and through the option Scale in Photoshop we converted the pixels to μm. Then using the ruler we measured the specific diameters and automatically the software made the conversion.

S.M.7 *Ex vivo* stimulation assays

10^6 DLN cells or TILs were isolated from the lungs of LLC-Ova-inoculated Control-, MTD-, MTR-, anti-PD-1- and MTR/anti-PD-1-treated mice and stimulated for 3 days in the presence of 75 μg/mL OVA (Sigma-Aldrich). In another set of experiments, 10^6 DLN cells or TILs were isolated from the lungs of B16-F10-inoculated Control-, MTD- or MTR-treated mice and stimulated for 3 days in the presence of 2 μg/mL soluble anti-CD3 and anti-CD28 antibodies (Biolegend).

S.M.8 Cytokine analysis

Cytokines were measured in cell culture supernatants using commercially available ELISA kits for mouse IFN-γ, TNF-α, GM-CSF and IL-10 (R&D Systems).
